# The Insane of Iowa

**Published:** 1853-10

**Authors:** D. L. McGugin


### IOWA MEDICAL JOURNAL.

VOL. I. KEOKUK, IOWA, OCTOBER, 1853. NO. 3.

### ORIGINAL COMMUNICATIONS.

### *The Insane of Iowa.* By Prof. D. L. McGugin.

An examination of the "Abstract of the Seventh Census" will show a population in the State of Iowa, of 192,214, including the free colored persons, of which number 40 only are reported to be insane.

The whole population of the U. S. is 23,191,673, of this number 15,768 are reported insane, or 1 in 1,470 of the inhabitants. If this proportion be general, then it would appear that there must be some error in the returns or in the mode of taking the Census. But the great disparity as shown in the returns from the different States is somewhat striking, and deserves especial notice. For instance in this State there is but 1 in 4,805 of our population, and in Maine there is 1 in 1088, in N. Hampshire 1 in 826, in Vermont 1 in 569, in Mass. 1 in 604, in Rhode Island 1 in 585.

To account for this disparity, we are left to suppose that the causes, in the production of this deplorable malady, are more abundant, more active, and more potent in some States than in others. In the N. England States the proportion is greater than in the Western States, and in the Northern than in the Southern. This is a subject of much importance and deserves attention and investigation.

Then first with regard to the great inequality in the results as found in the older, and in the new, or Western States. A peculiar fact in the history of insanity is, that the foreigner is peculiarly obnoxious to this dire affliction. The attention of Harvey L. Peet, L. L. D., has been called to it, and has given the subject much examination, and assigns as a reason for its increased frequency among them, that of "Home sickness, change of climate, and the various hardships of the emigrant's lot." No doubt is entertained whatever but that emotional influences, conjoined with physical derangements, do favor the production of insanity with all persons, and particularly with those in whom there is already a hereditary predisposition, and that where these causes exist most abundant, and occur most frequently, that there we would find unfortunately, cases of insanity multiplied. But let us enquire whether it would take place more readily with the foreigner who comes to this country, than those of our own citizens, who leave the older and eastern States, for localities in the West. The foreign emigrant, when he leaves his father land, leaves, it is true, his native land, endeared associates and long loved friends. But he consoles himself, at the same time, with the happy assurances that he has forsaken a land of oppression, of onerous exaction, ill-requited labor and effort, and finds refuge in a land of peace and plenty, where his labor will find a rich reward, and in which will be secured to him the cherished blessings of freedom and liberty. Here he is secure from oppression, never over-tasked, never over-worked; he is relieved from physical "hardships" and seldom wants for the comforts of life, provided he uses ordinary diligence. For all his toil, labor and effort, he receives ample remuneration and a compensation fully adequate to the wants and comforts of life. In this particular his condition is vastly improved, and therefore, his separation from the land of his nativity can occasion him but little sacrifice, save from the emotional influences above alluded to, namely those arising only from the fact of a separation from those to whom he was bound, by the ties of affinity and blood, in the domestic circle of his paternal home. He is anxious less to be *there*, than surrounded *here*, in his new asylum, by those endeared to him by associations and recollections of a peculiarly sacred character.

The emigrant, however, from the Eastern States, when he exchanges his birth-place for a new home in the West, suffers all the sacrifices which the foreign emigrant endures in the separation from his paternal roof, and the endeared circle of family and friends, but yet he has none of the solaces which the foreigner so richly enjoys.—He has sundered no tie which bound him to a country of oppression, he has no disgusts or cherished dislikes in retrospect, but has left his native State and every relation calculated to beget, and perpetuate, an abiding attachment. Lured by the glittering prospects of still greater success, he leaves a country of rich abundance, of easy acquirement, society in the highest condition of improvement, well known and long cherished friends, the graves of his ancestors, and plunges into the

"far west," where indeed he is often compelled to "*endure the hardships of the emigrant's lot,*" oftimes deprived of many of the necessary comforts of life, of society, and far distant from the familiar scenes of his childhood's mountain home;—if any where, or under any circumstances, "*home sickness*" would oppress the heart with unsatisfied and yearning desires,—here it would be felt and endured.

Superadded to this, he has suffered as great a change of climate as does the foreigner, for instead of the pure mountain air, to which from birth he has been accustomed, he is exposed to an atmosphere, which, at certain seasons, is rendered impure by miasmatic exhalations, from decaying timber in the deep forests and the rank vegetation of the unsubdued prairies, or the carboniferous exhalations from the exposed soil but recently upturned by the plough. Under these influences he is liable to be prostrated upon a sick couch, no old familiar friends or relatives, ready to administer to his wants,—to sympathise with or console him. With the standard of his health lowered, this self-abandonment, preys upon his mind and he sinks into despair or is lost in gloomy melancholy. His family too may fall under the prevailing atmospheric influences, and unable himself to extend a helping hand, we cannot in all our observation, conceive of causes of a mental or physical nature, more active or potent in dethroning the reason. The population of the new, is made up mainly by emigration from the older States, who in their efforts to subdue the forests, or the wide prairies, and bring them under the plastic hand of culture and improvement, these are they who suffer most from "*home sickness, the change of climate and the hardships of the emigrant's lot.*" If these last mentioned influences tend to swell the lists of insanity by the foreign emigration, of which we do not doubt, we can see no cogent reason why the western States should enjoy so happy an immunity from this mental malady, nor will we be convinced until the most reliable data are furnished us as proof.

Again, in the older States, where their systems are better matured, and those benevolent enterprises, the insane asylums, have received that attention demanded by the wants of this suffering class of our fellow men, the proportion of the insane, to the sane, is largely increased over the new States. This too is in the ratio of their duration; for in the group of N. England States, already given, the proportion of insane is largely increased over the other States, and yet their philanthropic citizens have long since made provision for their comfort and safety. And because of the deep interest, which has been awakened to their condition, and has been kept alive and perpetuated by the existence among them of these asylums, the enumeration is kept with more care and circumspection. In Ohio there is 1 in every 1,465 of its inhabitants, and here an institution has been in successful operation for the period of 15 years. Indiana has 1 in 1707, the asylum in this State has been not more than six years in existence. Illinois has 1 in 3,420, the asylum here has been in effective operation a shorter period than either of the former.

These facts at first view would indicate that the presence of asylums tended to an increase of insanity in those States in which they exist and that this increase is in the ratio of their duration. All experience shows that the very opposite is the case, for in well regulated and well managed institutions the *per cent.* of cures, in recent cases, is from 85 to 90. This furnishes another strong presumptive evidence amounting almost to positive proof, that the statistics on insanity are erroneous, for in those States where these benevolent enterprises exist, *there* the public attention has been awakened to the subject, resulting in the discovery of those who, before the establishment of these asylums, had been doomed, by neglect, to drag out their existence in a continued night of the mind.

There is another fact worthy of attention which goes far to strengthen this position. We will contrast the returns of those States in which these humane institutions have existed but for a short time, with those in which there is no provision for their recovery or safety. We have already referred to Indiana, where there is one in 1,707,—Illinois one in 3,420,—Missouri one in 2,418,—Tennessee one in 2,097. Here, in these States, public provision has been made for their relief, differing in the length of time since they were projected and completed. But in Wisconsin, where no institution exists, there is one in 6,362, and in Iowa, in the same unfortunate condition, there is one in 4,805.

Here then is another additional proof that where the attention of the humane and philanthropic is called to the subject, a much larger number of the unhappy afflicted is discovered, and that per consequence, in those States where no active investigation has been awakened, there are numbers in a state of suffering of whom nothing is known, beyond the family, to whom they are the continual objects of care and dread.

We do not wish to trespass upon the time or patience of the reader, but as the following facts bear so strongly upon the subject of the inaccuracy of our statistics that they are worthy of notice although they relate to another but kindred subject. Harvey L. Peet, L. L. D., in a paper read before the Medical Society of the State of New

York, in which he maintained that the enumeration of the deaf mutes was also imperfect, and attributes that imperfection to the want of a lively interest in the condition of that class of unfortunates, refers to the well known fact that in London, sixty years since when a proposition was made to found an asylum in that city for the benefit of the deaf mutes, the suggestion was regarded as a chimera, because no one had seen such an individual as one deaf and dumb, while, at that time, there were, in the city, several hundred. He also refers to the fact, that, thirty years ago in the city of New York, where a similar proposition was treated in a similar manner and for the same reasons, upon a close examination, however, as many as sixty-six were found in seven wards only.

In a further and more careful examination of the returns, some interesting facts present themselves with reference to the slaves of the South. If we would confide implicitly in the correctness of the returns in the Middle, Northern and Western States, we would find a ray of light beaming from an institution whose existence we are taught to deplore. We now refer to the very few cases of insanity as reported among the slaves of the South. In Texas there are 58,161 slaves; of this number *one only* is reported to be insane. There is a slave population in Mississippi of 309,878,—22 of these insane. In Alabama there are 342,872,—35 insane. Arkansas 47,100,—3 insane. Missouri 87,422;—9 insane. Except in one single instance, these results, with varying shades, would represent all the slave States.

Some questions of grave import will naturally arise in the mind from an examination of these facts as they present themselves in these reports. Is this exemption on the part of the slaves apparent only, or real? If apparent only, then there is even greater looseness and negligence in taking the enumeration than even in the North-Western States. Is the colored man, mentally and physically, so constituted as to give him this immunity? The low order of his mental condition would incline to dementia and we would for the same reason expect more cases of idiocy; therefore, in our opinion, we do not so often find these demonstrations of wild extravagance, in certain chronic cases, as are to be observed among the whites. And then freedom from care—from all anxiety of "the morrow," are circumstances which would go very far towards subduing all emotional influences. Mirth, too, is a prominent quality in their mental and moral organization, and their proneness to indulge in hilarity and merriment would greatly tend to sustain the mind from sinking into gloomy forebodings or helpless despair. How far their physical construction, particularly that of the brain or the disposition of its parts, would go towards securing them against a malady which would greatly aggravate the evils of their present condition, we shall not venture upon a hypothesis.

The remarkable exemption, as furnished in the case of Texas, where among so large a slave population there is but *one solitary* case of insanity, as reported, we fear would prove too much, and suffer from that law in logic which adjudges it to prove nothing at all. The climate does not *per se* exercise a protective Ægis, for the proportion of insane among the whites is much the same as in the Western States where the subject, as in many of the States of the South, has not engaged the public attention, or awakened philanthropy, and within whose limits no asylums exist. If there be any immunity with regard to the slaves, they bear within them mentally and physically, an inherent protective power. But let us examine this again and if this position be not sustained then we must fall back upon our original position; and that is, that, so far as reports go towards making up correct statistics, they are not reliable, but are calculated to mislead and injure.

If the colored man is endowed with the element of security and exemption, it will be found to be uniform under similar circumstances. We will call the reader's attention to the two Carolinas. In Carolina *South*, there are nearly 385,000 slaves, and only 9 of this number are insane. This instance, with the others enumerated, would prove powerful and overwhelming arguments in the hands of those who advocate the doctrine of an inherent mental or physical power protecting the slave, and an equally potent and convincing proof that united to this natural exemption, their domestic, civil, and social condition and relations, constitute the true reasons for it. Before we rush too hastily and rashly upon such conclusions we will pass into her sister State, Carolina *North*, where there is a less slave population, but the black man, as found in Carolina South, physically, mentally, morally, civilly and socially; and here are reported to be 24 insane in a slave population of 288,548, less 98,552 than in the former. Another fact. Of the population of *free* blacks in the United States, amounting to 433,-643, there are 321, or one in 1350, which is a larger average than with the whites. Now while we admit the force of the argument that the almost entire freedom from care, and that their mental and moral characteristics may, to a certain degree, save them from mental alienation; yet, we find the colored man liable to it under any and all circumstances, whether in the North or in the South, and whether bond or free.

We have referred to those influences and peculiarities which would favor an exemption among the slave population; but are there not some countervailing influences and circumstances of rather an aggravated character and which are unknown to our white population? Would not the separation of families, parents from children and children from parents, prove a strong emotional influence favoring its production. Are they as a race insensible apathists that such incidents unknown to the white population, would be unheeded and unfelt by them? This can be known only by observation, and the inquiry is made rather than an opinion given.

We have referred to the probability of an increase of idiocy and of dementia among the slaves because of the low standard of intelligence. This is indicated by the returns, which, if correct, would doubtless increase the result. Among the white population there are more insane than idiots, and yet, among the slaves there are nearly four idiots to one insane. Is it not then probable that many of those reported as idiots are not truly congenital cases, or rather that a majority of them are cases of dementia. If so, as this is one form of insanity, it goes to show that they are obnoxious to it, and that many cases reported as idiots should be classed among the insane.

After an examination of the whole subject we can arrive at no other conclusion than that, in this particular, the *vital statistics* of the country are unfortunately defective and inaccurate, and that steps should be taken to correct the evil. There is no fear that the enumeration will exceed the true and actual number in any case. It is an evil because when that enumeration is small the voice of humanity is stilled, benevolence is denied the exercise of its hallowed offices and patriotism is deprived of the vital element in its true purposes and functions, namely—the care, protection and preservation of those whom misfortune in its darkest aspects and moments has doomed to helplessness and woe.

